# Induction of atypical cell death in thyroid carcinoma cells by the indirubin derivative 7-bromoindirubin-3′-oxime (7BIO)

**DOI:** 10.1186/s12935-015-0251-8

**Published:** 2015-10-12

**Authors:** Martina Broecker-Preuss, Nina Becher-Boveleth, Susanne Gall, Katrin Rehmann, Susann Schenke, Klaus Mann

**Affiliations:** Division of Laboratory Research, Department of Endocrinology and Metabolism, University Hospital Essen, Hufelandstr. 55, Essen, Germany; Department of Clinical Chemistry, University Hospital Essen, Hufelandstr. 55, Essen, Germany; Clinic of Nuclear Medicine, University Hospital Essen, Hufelandstr. 55, Essen, Germany; Center of Endocrinology Alter Hof München, Dienerstr. 12, Munich, Germany

**Keywords:** Dedifferentiated thyroid carcinoma, Indirubins, 7BIO, Cell death

## Abstract

**Background:**

The indirubin derivative 7-bromoindirubin-3′-oxime (7BIO) has already shown anticancer properties by causing cell death in some tumour cell lines and may be a new therapeutic option for treatment-resistant tumour cells. Since dedifferentiated and anaplastic thyroid carcinomas do not take up radioiodine and are insensitive to chemotherapeutic treatment and external radiation, direct cell death induction in these tumour cells may be a promising approach. We thus investigated the effect of 7BIO on thyroid carcinoma cell lines of different histological origins and characterized the type of cell death induction by 7BIO.

**Methods:**

Cell viability was measured with MTT assay. Cell death was analysed by caspase 3/7 activity, lactate dehydrogenase liberation, caspase cleavage products, DNA fragmentation, cell cycle phase distribution and LC3B analysis.

**Results:**

After 7BIO treatment, cell viability was reduced in all 14 thyroid carcinoma cell lines investigated. Treated cells showed DNA fragmentation, cell cycle arrest and lactate dehydrogenase liberation but no LC3B cleavage. Caspase activation following 7BIO treatment was found in five of six cell lines investigated. Interestingly, inhibition of caspases had no effect on viability of the cells after 7BIO incubation.

**Conclusions:**

Our results indicate that 7BIO efficiently killed dedifferentiated thyroid carcinoma cells. It induced a non-classical kind of cell death that was caspase-independent and includes DNA fragmentation. 7BIO and related indirubin components thus may have value as a new therapeutic option for dedifferentiated thyroid cancer irrespective of the exact target molecules and the kind of cell death they induce.

## Background

The bis-indole alkaloid indirubin is the active compound of a traditional Chinese herbal medicine used for treatment of chronic myelocytic leukemia. Thus, indirubins are of interest as possible anti-tumour substances [1]. Indirubin and its analogues (referred to as indirubins) are naturally found in certain indigo-dye producing plants and sea shells [Review: 2], while various derivatives have been synthesized to improve the effects on tumour cells. Among the bromo-substituted indirubins, 7-bromoindirubin-3′-oxime (7BIO) showed activity against some tumour cell lines by inducing cell death [3–5]. While other indirubins were shown to inhibit cyclin-dependent kinases (CDKs), glycogen synthase kinase-3 (GSK-3), Janus activated kinases (JAK), SRC and subsequently STAT3 and AKT [6–11], 7BIO showed only marginal inhibitory effects towards these kinases but triggered a rapid cell death [3]. An inhibitory screening of 7BIO on 85 kinases was performed that revealed that only FLT3 was inhibited by 7BIO with an IC50 below 1 µM [3]. Despite the fact that the exact molecular mechanism of action of 7BIO is not yet known, it may be valuable for inducing cell death in tumour cells.

The imbalance between cell division and cell death pathways is one characteristic feature of cancer cells and contributes to the “hallmark of cancer cells” [12]. Decreased cell death in general is associated with hyperproliferative diseases like cancer and resistance to cell death contributes to uncontrolled tumour proliferation [13]. Cell death can be induced by different pathways that are regulated by different intracellular signalling cascades, i.e., mainly apoptosis, necrosis, necroptosis, and autophagy [Review: 14]. According to the Nomenclature Committee on Cell Death [15], apoptosis is a form of regulated cell death characterized by the activation of caspases, a family of cysteine proteases [16]. Caspase activation leads to a degradation of specific substrates, to a fragmentation of nuclear DNA, and, in turn, to characteristic morphological changes of the affected cells [17, 18]. Necrosis on the other hand is characterized by cell swelling and early plasma membrane permeabilisation followed by cell rupture and the release of cellular material [Review: 19]. Biochemically, lysosomal hydroxylases are often released during necrosis and are involved in cell destruction [20]. Autophagy, as the third type of cell death mechanism, describes the process of self-digestion of cellular components by the cells [Review: 21]. During this regulated cellular process organelles and cell components are degraded and recycled and thus, in addition to inducing death in single cells, it is also a survival mechanism for the whole cell population in situations like starvation or cellular damage [22].

Thyroid carcinoma is the most common endocrine malignancy, accounting for nearly 95 % of malignant endocrine tumours [23]. Most thyroid carcinomas (95–97 %) originate from follicular thyroid cells, whereas about 3–5 % of thyroid carcinomas are medullary subtypes derived from C-cells [24]. About 90 % of the thyroid cancers of follicular cell origin are well differentiated papillary thyroid carcinoma (PTC; 70–80 %) or follicular thyroid carcinoma (FTC; 10–20 %). While differentiated subtypes still take up radioiodine and have a good prognosis, initially 10 % or less of thyroid carcinomas are poorly differentiated or undifferentiated, anaplastic subtypes [24, 25]. Loss of differentiation includes loss of radioiodine uptake and storage and an aggressive and infiltrative growth pattern. Thus, dedifferentiated and anaplastic thyroid carcinomas (ATC) are tumours that are insensitive to radioiodine treatment, chemotherapeutic treatment, and external radiation and have a bad prognosis [26, 27]. A subgroup of patients with differentiated PTC or FTC also get recurrent disease even under TSH suppressive therapy, even though most PTC and FTC patients are successfully treated with surgery and subsequent radioiodine treatment to destroy residual tumour cells [23, 26]. Patients with recurrent disease have different outcomes due to the degree of dedifferentiation of their tumour and due to its ability to take up radioiodine [26, 28]. Chemotherapeutic treatment of dedifferentiated or anaplastic thyroid carcinoma is less effective with partial response rates of 25 % or less [29, 30]. Since most cytotoxic drugs kill cells by inducing cell death pathways [14], the resistance of thyroid carcinoma cells towards these drugs may point to the cells’ reduced ability to undergo cell death. Thus, for dedifferentiated and anaplastic thyroid cancer, facilitating cell death induction is one new therapeutic option. Since 7BIO had already shown anti-tumour activities in other cell models, we performed this study to evaluate the effectiveness of 7BIO in thyroid carcinoma cells.

## Methods

### Compounds and antibodies

7BIO came from Enzo Life Sciences (Farmingdale, NY, USA), 3-methyladenine (3-MA) and *N*-(2-Quinolyl)valyl-aspartyl-(2,6-difluorophenoxy)methyl ketone (Q-VD-OPh) were provided by Millipore/Calbiochem (MA, USA), obatoclax and staurosporine were from Selleck Chemicals (Houston, TX, USA). All compounds were dissolved in DMSO to 5–10 mM, stored in aliquots at −20 °C and further diluted in the appropriate medium. Microtubule-associated protein 1A/1B-light chain B (LC3B) antibodies were from Cell Signaling Technology (Danvers, MA, USA).

### Cell lines and cell culture

Cell lines from different subtypes of thyroid cancer were used in this study: SW1736 [31], HTh7 [32], C643 [33], HTh74 [34], 8305C [35] and 8505C [36] were derived from ATC. BHT101 [37], B-CPAP [38] and TPC1 [39] were from PTC. ML1 [40], TT2609 [41], FTC133 [42], FTC236 [42] and FTC238 [42] were derived from FTCs. The FTC133, FTC236 and FTC238 cell lines [42] were derived from a single primary FTC, a lymph node metastasis and a lung metastasis from the same patient. Jurkat leukemia cells and HepG2 hepatocellular carcinoma cells were used as controls. SW1736, HTh7, HTh74 and C643 cells were kindly provided by Prof. Heldin (Uppsala, Sweden), the other cell lines were purchased from ATCC (Manassas, Virginia, USA), ECACC (Salisbury, UK) and DSMZ (Braunschweig, Germany).All cells were grown in their appropriate medium supplemented with 10 % foetal bovine serum (FBS; Life Technologies, Paisley, PA) at 37 °C at 5 % CO_2_.

### Cell proliferation studies

For the proliferation assays, 1 × 10^4^–5 × 10^4^ cells (depending on the cell line) were seeded in each well of a 96 well plate. After 24 h, medium was removed and culture medium without FBS containing 0.1 % bovine serum albumin (BSA) and the concentrations of 7BIO, Q-VD-OPh, 3-MA or a combination of 7BIO and Q-VD-OPh or 3-MA as indicated or vehicle was added. After 48 h, viable cells were stained with the Cell Titer Aqueous One Solution assay for 2–3 h (Promega, Madison, WI, USA) and optical density at 490 nm was read using an Emax microplate photometer (Molecular Devices, Sunnyvale, CA, USA). Control values without stimulation were performed as 22 fold determinations, while all concentrations of 7BIO, Q-VD-OPh and 3-MA were tested in eightfold. The results and two-tailed Student’s t tests were calculated with SoftMax pro software (Molecular Devices). The IC50 values (drug concentration that caused a 50 % reduction in MTT assay) were calculated with four parameter logistic function dose–response curves using Sigma Plot software (Systat, San Jose, CA, USA).

### Determination of LDH release and caspase 3/7 activity measurement

The CytoTox-ONE homogeneous membrane integrity assay (Promega) was used to measure the release of lactate dehydrogenase (LDH) from damaged cells. Activity of caspases 3 and 7 was measured by the Apo-ONE homogeneous Caspase-3/7 assay (Promega). For both assays, cells were seeded and grown as described above, except that black 96 clear bottom well plates were used. On day 2, medium was removed and 100 µl culture medium with 0.1 % BSA containing 3 µM 7BIO was added to each well. After 24 h, 50 µl of medium from each well was transferred to a second 96 well plate and equilibrated to 20 °C. 50 µl of CytoTox reagent was added and the solution was incubated for 10 min in the dark at room temperature. After adding 25 µl of stop solution, fluorescence was determined with an excitation wavelength of 560 nm and an emission wavelength of 590 nm. In each experiment, controls without cells and fully lysed cells as maximum LDH release controls were included. Caspase 3 and 7 activity in 7BIO-treated cells was determined in the original stimulation plate by adding of 50 µl of Apo-ONE reagent that contained a fluorometric caspase substrate, cell lysis reagent and buffer. For positive controls, Jurkat cells that grow in suspension culture were harvested by centrifugation, resuspended in medium with 0.1 % BSA, seeded and staurosporine or a combination of staurosporine and Q-VD-OPh was added. After 24 h, 96 well plate was centrifuged, 50 µl of supernatant was discarded and Apo-ONE assay was performed. After 60 min, fluorescence was measured at 521 nm (emission) after excitation with 499 nm. All values were determined as eightfold determinations. Calculation of results and two-tailed Student’s t tests were performed using SoftMax pro software (Molecular Devices).

### Cell cycle analysis

To perform cell cycle analyses, cells were plated in 6 well plates (1 × 10^5^–5 × 10^5^ cells per well) in their appropriate growth medium. After 24 h, medium was removed and medium without FBS containing 0.1 % BSA and 3 µM 7BIO was added for the indicated times. Cells were harvested and fixed in ice cold 70 % ethanol. RNase A (60 µg/ml) and propidium iodide (25 µg/ml) in PBS were added and samples were incubated for 20 min at room temperature in the dark. The cellular DNA content was measured by means of a FACS Calibur flow cytometer (Becton–Dickinson, San Jose, CA) and the cell cycle stages and subG1 peak were analysed by ModFit Software (Verity Software House, Topsham, ME, USA).

### Cell stimulation and protein extraction

For the ELISA and western blot analyses, cells were seeded on cell culture dishes (15 cm diameter) and grown for 1–2 days until they reached 80–85 % confluence. Full medium was replaced with medium containing 0.1 % BSA and cells were maintained in this medium for 1 h before 3 µM 7BIO or vehicle was added. Non-adherent Jurkat cells were harvested by centrifugation, resuspended in medium with 0.1 % BSA and staurosporine or a combination of staurosporine and Q-VD-OPh was added, respectively. After the indicated stimulation times, the medium was removed and the cells were washed with ice-cold PBS. All additional steps were performed on ice. For the cell lysis, a lysis buffer containing protease and phosphatase inhibitors (Complete protease inhibitor and phosStop phosphatase inhibitor, Roche Applied Science, Mannheim, Germany) was used. The lysates were clarified by centrifugation at 10,000×*g* for 10 min at 4 °C. The protein concentration was determined with a modified Bradford assay (Bio-Rad Laboratories, Hercules, CA, USA).

### Cleaved caspase and cleaved PARP ELISA

A semi-quantitative determination of cleaved caspase 3 (Asp175) and cleaved poly (ADP ribose) polymerase (PARP) as a marker of apoptosis induction and protease activation was performed by using specific sandwich ELISAs for these cleaved proteins (Cell Signaling Technologies). In brief, cells were plated, stimulated, and lysed as described above. 100 µl of diluted cell lysate containing 100 µg of total cell protein was incubated in each of the antibody coated well of the plate overnight at 4 °C. After washing, we used an antibody specific for the cleaved protein and a HRP-labelled secondary antibody for detection. The TMB substrate reaction was stopped after 30 min at room temperature and the absorbance was measured at 450 nm (EMax microplate reader). The results were calculated as percent of unstimulated controls using SoftMax pro software (Molecular Devices).

### Western blot analyses

Western blot analyses were performed to analyse the effects of 7BIO on LC3B cleavage. 30 µg of total protein from vehicle stimulated and stimulated cells (see above) were denatured by boiling for 5 min in SDS sample buffer. Proteins were separated by SDS-PAGE on stain-free polyacryl-amide gels (Bio-Rad Laboratories) to enable loading control. After electrophoresis, optical densities of stained proteins in each lane were documented with a CCD camera system and verified using the Quantity One software (both Bio-Rad Laboratories). When the integrated optical densities of proteins in each lane did not differ more than 10 %, proteins were transferred to a nitrocellulose membrane (Bio-Rad Laboratories). After blocking with BSA, the blots were incubated with the LC3B primary antibody (Cell Signaling Technologies) in TBS containing 0.1 % Triton X100 overnight at 4 °C. After washing, an appropriate secondary antibody coupled to horseradish peroxidase was added. Detection of bound antigens was performed by an enhanced chemiluminescence detection kit (Amersham ECL Advance, GE Healthcare, Piscataway, NJ, USA). Signal intensity was evaluated with a CCD-camera (Bio-Rad Laboratories).

## Results

### Inhibition of proliferation after 7BIO treatment

**Fig. 1:**
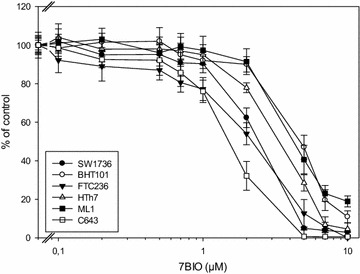
Decreased viability of thyroid carcinoma cells after 7BIO incubation. Cells were cultured in the presence of increasing concentrations of 7BIO or vehicle control for 48 h and viability was assessed by MTT assay. Values represent percent of vehicle control, mean ± standard deviation from eight-fold determinations. IC50 values are shown in Table 1

14 thyroid cell lines derived from follicular, papillary and anaplastic thyroid carcinomas were treated with increasing concentrations of 7BIO or vehicle for 48 h. For all cell lines, IC50 values measured by MTT assay are shown in Table 1. As examples, results for six cell lines are shown in Fig. 1; one data point represents the mean of eight values ± standard deviation. We found IC50 values for 7BIO in a similar range for all cell lines examined independent of the subtype of thyroid carcinoma they were derived from (1.54–4.83 µM). C643 anaplastic thyroid carcinoma cells had the lowest IC50 value (1.54 µM) while BHT101 cells (dedifferentiated papillary thyroid carcinoma cell line) had the highest IC50 value for 7BIO (Table 1) with 4.83 µM, respectively. These results indicate that 7BIO is effective in reducing the number of viable thyroid carcinoma cells in cell lines derived from various thyroid carcinoma subtypes.

**Table 1:** IC50 values of thyroid carcinoma cell lines after 48 h of treatment with 7BIO (MTT assay)

Cell line	Origin	IC50 7BIO (µM)
FTC133	Follicular	1.98 ± 0.32
FTC236	Follicular	2.21 ± 0.30
FTC238	Follicular	2.17 ± 0.27
ML1	Follicular	4.27 ± 0.38
TT2609	Follicular	4.62 ± 0.51
BHT101	Papillary	4.83 ± 0.36
B-CPAP	Papillary	4.73 ± 0.40
TPC1	Papillary	4.68 ± 0.37
SW1736	Anaplastic	2.52 ± 0.18
HTh7	Anaplastic	3.08 ± 0.25
C643	Anaplastic	1.54 ± 0.21
HTh74	Anaplastic	2.06 ± 0.18
8305C	Anaplastic	3.30 ± 0.27
8505C	Anaplastic	4.32 ± 0.41

### Cell cycle analyses after 7BIO treatment

**Fig. 2:**
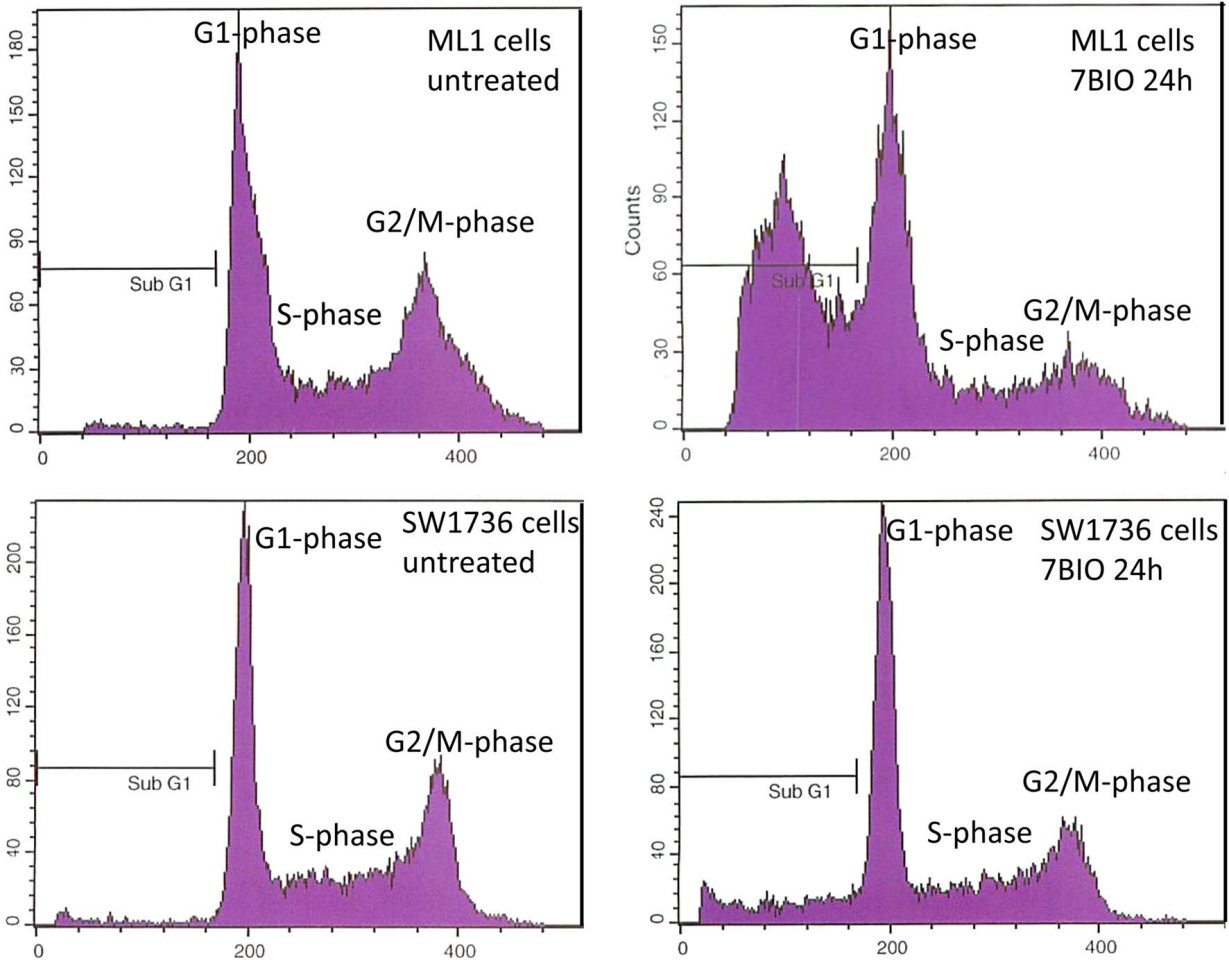
Cell cycle changes in ML1 and SW1736 cells after incubation with 3 µM 7BIO for 24 h. Cell cycle analysis was conducted using FACS, results for ML1 and SW1736 cells are shown as examples. Besides the increase in subG1 peak, in the remaining living cells an increase in G1 phase and a decrease in S phase were observed. Values for all cell lines examined are shown in Table 2

Cell cycle analyses and the following experiments to determine the kind of cell death caused by 7BIO were performed in the following six cell lines: FTC236 (FTC), ML1 (FTC), BHT101 (PTC), SW1736 (ATC), HTh7 (ATC), and C643 (ATC). Cell cycle analyses of the propidium iodide stained cellular DNA after a 24 h treatment with 3 µM 7BIO showed a marked increase of cells in subG1 fraction in all cell lines analysed, pointing to cell death and DNA fragmentation induced by 7BIO treatment (Table 2; Fig. 2). Interestingly, the fraction of cells in subG1 fraction was the highest in the two follicular cell lines FTC236 and ML1 (43.6 and 44.0 % of cells measured vs. 9.3–13.1 % in the other cell lines; Table 2). In the remaining living cells, the portion of cells in the G1 phase of the cell cycle was significantly increased after incubation with 7BIO in all cell lines except for C643 which exhibited a slight, but not significant decrease (Table 2). The portion of cells in the G2/M phase and/or the S phase of the cell cycle was diminished by 7BIO in all six cell lines investigated, although in different ways: while ML1 and BHT101 showed a decrease in both G2/M and S phases, SW1736 and HTh7 depicted only a decrease in S phase. FTC238 had a reduction only in G2/M phase and C643 showed an increase of cells in the S phase after 7BIO treatment, while the portion of cells in G2/M phase was significantly diminished (Table 2).

**Table 2:** Distribution of cell cycle phases in vehicle-treated and 7BIO-treated cells (24 h, 3 µM)

Cell line	Type	Status	% subG1	% G1	% G2/M	% S
FTC236	Follicular	Untreated	0.2 ± 0.1	38.4 ± 4.3	18.7 ± 0.8	42.9 ± 3.0
		7BIO 24 h	43.6 ± 4.7*	48.7 ± 4.0*	6.2 ± 0.9*	45.1 ± 5.1
ML1	Follicular	Untreated	1.2 ± 0.2	39.4 ± 5.0	29.5 ± 2.4	31.1 ± 3.5
		7BIO 24 h	44.0 ± 5.3*	76.8 ± 9.3*	16.9 ± 1.5*	6.3 ± 0.7*
BHT101	Papillary	Untreated	0.5 ± 0.2	51.3 ± 4.3	17.4 ± 1.1	31.4 ± 1.5
		7BIO 24 h	11.5 ± 2.1*	83.6 ± 6.8*	10.4 ± 0.7*	6.0 ± 1.1*
SW1736	Anaplastic	Untreated	1.2 ± 0.2	36.4 ± 3.7	22.1 ± 1.8	41.5 ± 2.4
		7BIO 24 h	12.7 ± 1.6*	48.2 ± 6.1*	18.5 ± 3.4	33.3 ± 3.5*
HTh7	Anaplastic	Untreated	1.0 ± 0.1	52.0 ± 6.4	15.1 ± 1.3	32.9 ± 2.8
		7BIO 24 h	13.1 ± 1.5*	63.3 ± 3.9*	17.3 ± 1.5	19.4 ± 0.9*
C643	Anaplastic	Untreated	0.6 ± 0.1	53.8 ± 6.7	12.4 ± 0.7	33.7 ± 1.2
		7BIO 24 h	9.3 ± 1.1*	48.7 ± 6.8	5.5 ± 0.3*	45.9 ± 4.5*

Values for G1-, G2/M- and S-phase are determined for the living cells that were not included in the sub-G1-peak

* Significant change (p < 0.05, Student’s t test) compared to controls treated with vehicle

### Cell death after 7BIO treatment

**Fig. 3:**
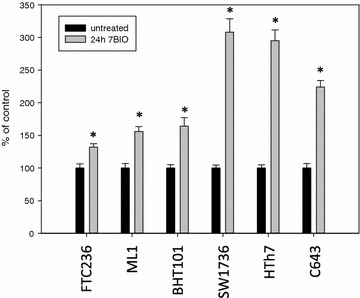
Increased LDH-release into the cell culture medium after treatment with 3 µM 7BIO for 24 h. Values were determined by CytoTox-assay and are depicted as percent of vehicle-treated control, mean ± standard deviation from eight-fold determinations; *asterisk* indicates significant increase (p < 0.05, Student’s t test)

Cells treated with 7BIO were analysed biochemically to evaluate the kind of cell death induced by this treatment. Figure 3 shows the LDH activity in supernatants of vehicle treated cells and cells incubated with 3 µM 7BIO for 24 h. All cell lines had a significantly elevated LDH content in the stimulation medium after 24 h of stimulation. LDH release in anaplastic SW1736, HTh7 and C643 cells was significantly higher than in follicular and papillary cell lines following 7BIO treatment. Elevated LDH activity in the cell culture medium pointed to a disruption of cell membranes with a release of LDH and other cytoplasmic components and thus reflected thyroid carcinoma cell disruption after the 7BIO treatment.

**Fig. 4:**
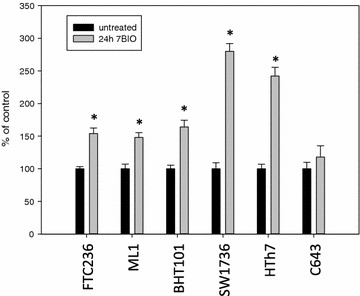
Increased activity of caspase 3 and 7 in thyroid carcinoma cell lines treated with 3 µM 7BIO for 24 h. Values were determined by the ApoOne assay, mean ± standard deviation from eight-fold determinations; *asterisk* indicates significant increase (p < 0.05, Student’s t test) compared to controls treated with vehicle

**Fig. 5:**
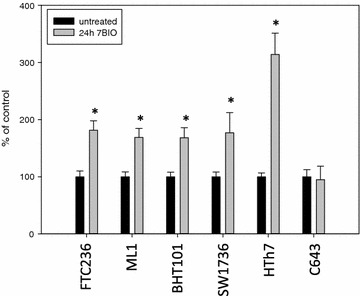
Increased concentration of cleaved caspase 3 fragments in protein extracts of thyroid carcinoma cells treated with 3 µM 7BIO for 24 h. Results of an ELISA specific for caspase 3 fragments are depicted, mean ± standard deviation from four-fold determinations; *asterisk* indicates significant increase (p < 0.05, Student’s t test) compared to controls treated with vehicle

**Fig. 6:**
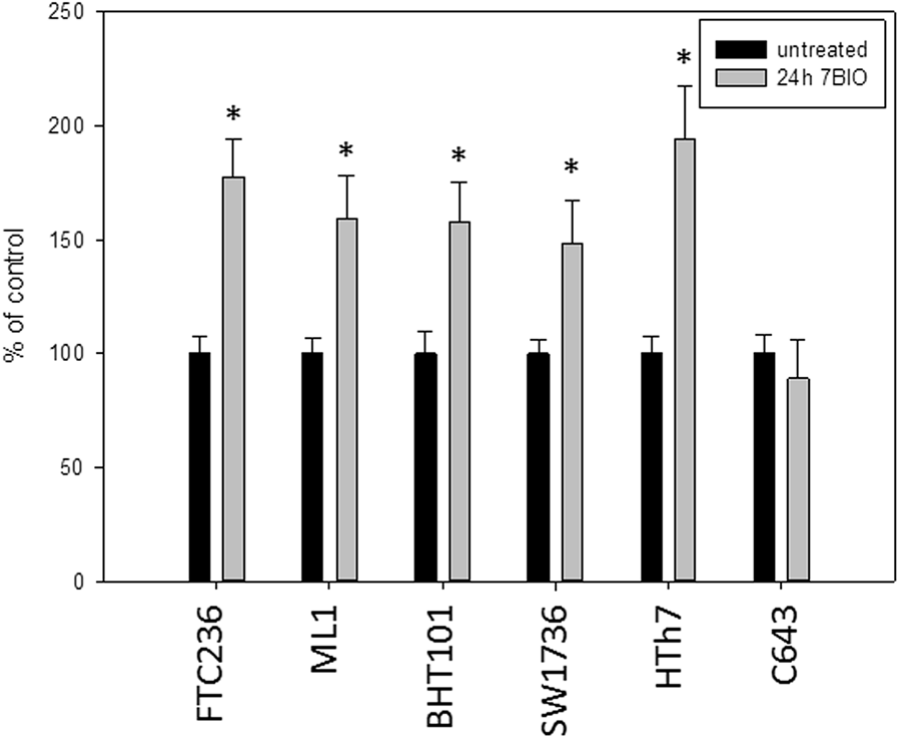
Increased concentration of cleaved PARP fragments in protein extracts of thyroid carcinoma cells treated with 3 µM 7BIO for 24 h. Results of an ELISA specific for PARP fragments are depicted, mean ± standard deviations from four-fold determinations; *asterisk* indicates significant increase (p < 0.05, Student’s t test) compared to controls treated with vehicle

Caspase 3 and 7 measurements as well as the determination of the cleaved caspase and cleaved PARP were performed to investigate apoptotic cell death mechanisms after the 7BIO treatment in thyroid carcinoma cells. As shown in Fig. 4, caspase activities were significantly elevated after 24 h of 7BIO treatment in all thyroid carcinoma cell lines except C643. FTC236, ML1 and BHT101 cells showed a relatively small, but significant increase in caspase activity after 7BIO treatment, while SW1736 and HTh7 cells depicted a higher caspase activity after incubation with 7BIO. Concentrations of cleaved caspase 3 and cleaved PARP as biochemical markers of activated caspases were also significantly elevated in all treated cells lines except C643 (Figs. 5, 6) pointing to an activation of the apoptosis machinery in these five cell lines caused by the treatment with 7BIO. However, the caspase activities depicted by our thyroid carcinoma cells were not as high as expected since with the concentration of 3 µM 7BIO, approximately half of the cells were dying (Fig. 1 and controlled by microscopy; data not shown). As positive controls for apoptosis induction, Jurkat cells were incubated with 2.5 µM staurosporine. As already described in literature [43], this treatment caused apoptosis with caspase activation (952 ± 103 % of untreated control), as well as an increase in cleaved caspase (1462 ± 98 % of untreated control) and cleaved PARP (1271 ± 116 % of untreated control). We performed co-incubation of thyroid carcinoma cell lines with 7BIO and the general caspase inhibitor Q-VD-OPh and measured viability after 48 h. As shown in Table 3, co-incubation did not increase the viability of the cells indicating that the activation of caspases is not the determining step in cell death execution but plays a less important role in 7BIO induced cell death of the thyroid carcinoma cells examined in this study. As positive control for Q-VD-OPh treatment, co-incubation of Jurkat cells with staurosporine and Q-VD-OPh resulted in a decrease of apoptosis induction by staurosporine (caspase activity 135 ± 19 % of untreated control; cleaved casapse 144 ± 15 % of untreated control; cleaved PARP 138 ± 14 % of untreated control).

**Table 3:** Viability of cells after incubation with 3 µM 7BIO, 5 µM Q-VD-OPh, 2 µM 3-MA or the combinations indicated for 24 h (MTT assay)

Cell line	Origin	7BIO	Q-VD-OPh	7BIO + Q-VD-OPh	3-MA	7BIO + 3-MA
FTC236	Follicular	41.5 ± 4.8*	102.4 ± 6.9	45.2 ± 7.2	94.6 ± 10.4	39.3 ± 7.8
ML1	Follicular	72.3 ± 4.2*	105.1 ± 6.0	70.3 ± 6.8	106.0 ± 7.5	71.6 ± 7.7
BHT101	Papillary	68.3 ± 8.3*	97.4 ± 8.2	71.2 ± 5.3	98.4 ± 5.7	69.4 ± 9.4
SW1736	Anaplastic	39.5 ± 8.2*	95.7 ± 11.4	44.5 ± 9.1	104.6 ± 9.7	42.6 ± 9.5
HTh7	Anaplastic	50.8 ± 6.2*	100.6 ± 7.9	53.5 ± 8.3	106.7 ± 10.3	49.2 ± 7.5
C643	Anaplastic	21.5 ± 4.8*	104.5 ± 9.7	22.9 ± 5.5	99.1 ± 6.9	18.6 ± 7.4

* Significant decrease (p < 0.05, Student’s t test) of 7BIO-treated cells compared to vehicle-treated controls, while treatment with Q-VD-OPh or 3-MA alone had no significant effect compared to vehicle-treated controls. Treatment with a combination of 7BIO and Q-VD-OPh or 7BIO and 3-MA was compared with the 7BIO treatment and had no significant effects

**Fig. 7:**
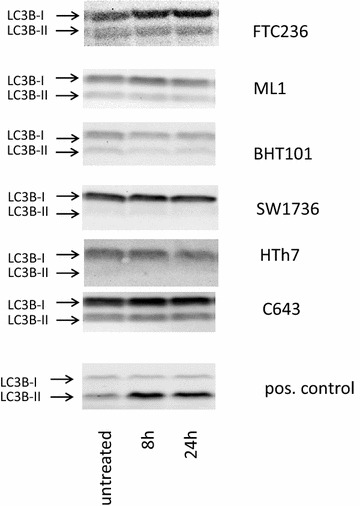
Absence of conversion of LC3B-I after treatment with 3 µM 7BIO indicating no involvement of autophagic processes in 7BIO-mediated cell death. Western blot analyses of vehicle-treated and 7BIO-treated thyroid carcinoma cell lines using LC3B antibody. As a positive control for autophagy induction, HepG2 cells treated with 0.2 µM obatoclax were included. Equal protein loading was ensured by in-gel protein staining (see “Methods”)

Finally, LC3B conversion as a marker of autophagic cell death was examined by western blot analyses after 7BIO treatment. In all six thyroid carcinoma cells examined, 7BIO treatment did not result in LC3B cleavage which indicates that autophagic processes are not involved in cell death by 7BIO in thyroid carcinoma cells (Fig. 7). HepG2 cells treated with obatoclax, a BH3 mimetic that was already reported to cause autophagy and LC3B conversion [44] were used as positive control for these experiments and depicted a clear conversion of LC3B isoforms as expected (Fig. 7). Moreover, co-incubation with 7BIO and 3-MA, an autophagy-inhibitor, did not result in an increased viability of cells, which again speaks against an involvement of autophagic processes in 7BIO induced cell death (Table 3).

## Discussion

In this study we showed the efficacy of the indirubin derivative 7BIO in inducing cell death in thyroid carcinoma cell lines. The kind of cell death induced was a non-classical death with cell cycle block, DNA fragmentation as well as signs of non-classical apoptosis and necrosis.

The direct pharmacological induction of cell death is one possibility to overcome the resistance of cancer cells towards apoptosis that is a hallmark of tumour cells [12]. Dedifferentiated and anaplastic thyroid carcinoma cells that have lost the ability to take up radioiodine are tumour cells that are resistant to cell death induction by external radiation and chemotherapeutic treatment [23]. One reason for this resistance may be the inability of these cells to undergo apoptotic cell death [14]. Direct induction of cell death therefore presents one possibility of new treatment strategies for these tumours. 7BIO is an indirubin derivative that has been recently described as inducing cell death in some tumour cell lines of various origins [3–5]. In this study we showed that 7BIO is also active against dedifferentiated thyroid carcinoma cells.

7BIO showed inhibitory effects on all 14 thyroid carcinoma cell lines investigated. IC50 values of our cell lines were in a narrow range (1.54–4.83 µM) with the C643 ATC cell line being the most sensitive cell line (IC50 of 1.54 µM; Table 1). All three papillary cell lines had IC50 values in the higher range (4.68–4.83 µM; Table 1) with BHT101 showing the highest IC50 value of our cell lines. In FTC and ATC cell lines, no correlation was found between the 7BIO sensitivity and the thyroid carcinoma subtype from which the cell lines had been derived (IC50 values in FTC cell lines 1.98–4.62 µM; IC50 in ATC cell lines 1.54–4.32 µM; Table 1). The molecular reasons for these differences have to be elucidated and may be due to specific activation of signaling pathways in these histological thyroid carcinoma subtypes. Moreover, the IC50 values in thyroid carcinoma cells were in the lower range of that reported in 7BIO-treated cell lines of other tumours (2.3–20.0 µM for breast cancer cell lines and approx. 12 µM for SH–SY5Y neuroblastoma cells) [3, 5] After incubation with 10 µM 7BIO for 48 h, all cell lines except ML1 achieved values of less than 10 % of control pointing to cell death in the majority of cells. A similar effect was already described by Ribas et al. [4] who showed that a 24 h treatment with 25 µM 7BIO was lethal for the entire cell population of SH-SY5Y neuroblastoma cells and Jurkat leukaemia cells. Our biochemical data in six 7BIO-treated thyroid carcinoma cells argue for an atypical kind of cell death with biochemical signs of necrosis, DNA fragmentation, cell cycle inhibition, and atypical apoptosis. An increased LDH release typical for cell death by necrosis or secondary necrosis was seen in all six cell lines examined after 7BIO stimulation. In parallel, a small but significant increase in activity of caspases seen as direct caspase activity and increase in caspase cleavage products was found in all cells but C643. However, the significance of apoptosis-induction for cell death in 7BIO stimulated cells has to be questioned since the pan-caspase inhibitor Q-VD-OPh did not significantly prevent cell death by 7BIO (Table 3). Similar results have already been described in other cell lines for 7BIO. In neuroblastoma cells and the T cell leukemia cell line Jurkat, cell death by an apoptosis-independent manner was induced by 7BIO [3, 4]. In IMR-5, IMR-32, Jurkat and HL-60 cells, 7BIO treatment killed cells without activating caspases [3, 45]. Nicolaou et al. [5] on the other hand reported on 7BIO-mediated apoptosis induction by caspase-dependent as well as by caspase-independent pathways in breast cancer cell lines with an increase in the uncleaved caspases 3 and 9. From their own data and data in the literature these authors concluded that the response of different tumour cell lines to 7BIO through apoptotic or non-apoptotic mechanisms is a function of cell content [5]. Our own data suggest a cell death mechanism in which caspase activation occurs but is of little importance. This thesis is supported by the C643 anaplastic thyroid carcinoma cell line that was the most sensitive cell line towards 7BIO incubation but showed no significant activation of caspases by 7BIO. Caspase-independent cell death by apoptosis has already been described in various cell systems [46]. Physiologically it occurs in cells expressing endogenous caspase inhibitors like XIAP, CrmA or AIF [47–50]. In affected cells, apoptosis-like death is generally caused by serine-protease-mediated processes [51] that mediate the cleavage of cellular substrates similar to caspase-induction. The lysosomal proteases cathepsin D and B as well as calpains and other serine-proteases and are involved in this caspase-independent, apoptosis-like cell death [52–55]. The findings on the kind of cell death in our 7BIO-stimulated thyroid carcinoma cell lines are in accordance with these findings in cell lines undergoing cell death after various stimuli and also with the recently reported results of Ribas et al. [4] who reported on the involvement of serine proteases in 7BIO-induced cell death. Further experiments to analyse protease activation and to characterize intracellular targets of 7BIO may elucidate the exact kind of cell death induced by 7BIO.

Regarding increases in subG1 fraction after 7BIO treatment, follicular FTC236 and ML1 cells interestingly showed a large fraction of cell in subG1 peak pointing to massive DNA fragmentation in these cells like already described by others [5]. In the other cell lines examined, fractions of cells in subG1 fraction were also significantly elevated, but to a lesser extent. Since DNA fragmentation can be mediated by various proteolytic enzymes, a caspase-independent mechanism of DNA fragmentation probably occurred with different degrees of activation in different 7BIO-treated thyroid carcinoma cell lines. In other cell lines, a caspase-independent, serine protease mediated DNA cleavage has already been described [51, 56, 57]. However, DNA fragmentation resulting in an elevated subG1 fraction may also occur during necrotic cell death [58] and in our cells may reflect a mixed kind of cell death with non-classical apoptosis and necrotic features. This interpretation is in accordance with data from the literature that cell death in vivo is often characterized not by a single cell death mode but can comprise elements of apoptotic, necrotic, and sometimes autophagic elements. Accordingly, various kinds of cell death can occur independently of each other or simultaneously resulting in a combined cell death phenotype [59, 60]. Cell cycle analyses of the remaining living cells pointed to different regulation mechanisms in various thyroid carcinoma cell lines treated with 7BIO. All cell lines except C643 showed a significant increase in the portion of cell in G1 fraction after incubation with 7BIO. This is in contrast to some other cell lines described in the literature that exhibited a decrease in G1 fraction following 7BIO treatment [3, 5] that is also described for other indirubins [6, 61, 62]. C643 cells showed a similar cell cycle distribution with a small, but not significant decrease in G1 phase (Table 2). The activation of different checkpoints of the cell cycle points to different cellular backgrounds of cells with respect to cell cycle regulatory proteins like p53, pRb as well as CDKs and its regulatory proteins [63]. Further studies may reveal the correlation of expression pattern of cell cycle regulatory proteins with the cellular effects of 7BIO treatment in different cells.

## Conclusion

In summary, we found that 7BIO efficiently killed dedifferentiated and anaplastic thyroid carcinoma although its exact mechanism of action and the exact kind of cell death induced by 7BIO is not yet known and needs further investigation. Nevertheless, 7BIO and related indirubin components like 6BIO and other recently described indirubin derivatives [7–11] may have value as a new therapeutic option for dedifferentiated thyroid cancer.

## Abbreviations

3-MA: 3-methyladenine
7BIO: 7-bromoindirubin-3′-oxime
AIF: apoptosis-inducing factor
ATC: anaplastic thyroid carcinoma
CDK: cyclin-dependent kinase
CrmA: cytokine response modifier
FTC: follicular thyroid carcinoma
GSK-3: glycogen synthase kinase-3
JAK: Janus activated kinases
LDH: lactate dehydrogenase
PARP: poly-(ADP-ribose)-polymerase
PDTC: poorly diferentiated thyroid carcinoma
PTC: papillary thyroid carcinoma
Q-VD-OPh: *N*-(2-Quinolyl)valyl-aspartyl-(2,6-difluorophenoxy)methyl ketone
XIAP: X-linked inhibitor of apoptosis protein
